# Montreal Veterinary Medical Association

**Published:** 1897-02

**Authors:** B. A. Sugden

**Affiliations:** Secretary


Secretary.

### MONTREAL VETERINARY MEDICAL ASSOCIATION.

A MEETING of the Society was held on December 17, 1896, when the chair was occupied by Prof. Mills.

Mr. Connelley reported a case of "Entropium on the Lower Eyelid of a Fox-hound Bitch." The eye presented a very unsightly appearance, and the animal seemed to suffer a great deal of pain; therefore, at the request of the owner, Mr. Connelley decided to operate, which he did in the following manner: After removing the hair from the part it was well washed with a boric-acid solution, and a 4 per cent. solution of cocaine injected. Mr. Connelley then removed an elliptical piece of skin from the affected lid,and, drawing down the portion in contact with the eye, inserted three stitches, thus bringing the lid into its normal position. The wound was dressed with boric acid and iodoform, and in fifteen days had entirely healed, with the result that the bitch made an excellent recovery.

After complimenting Mr. Connelley upon the success of his operation the chairman called upon Mr. Sugden for his paper on "Pneumonia." After describing the various forms of pneumonia and their pathological appearances, the essayist reported a case which had recently occurred in the college practice and terminated fatally. A full report of the post-mortem appearances, which pointed to lobar pneumonia and acute septicæmia, was given, and of the bacteriological examination, which revealed a general infection by strepto- and staphylococci. The essayist briefly referred to the work done by the brothers Klemperer on the subject of immunity from this disease, and in closing thanked Dr. Martin and Mr. Hammond (Pathological Laboratory, McGill University) for the assistance they had so kindly rendered him. After some discussion by the members on the subject of counter-irritation Dr. Baker and Dr. Martin made some instructive remarks upon the use of aconite in this disease. Before closing the meeting the chairman briefly referred to his experience of pneumonia in dogs.

A regular meeting was held January 14, 1897, the chair being taken by Dr. Charles McEachran.

As there was no business to come before the Society, the chairman called upon Mr. Moore to report his case. This was one of "Acute Indigestion," which occurred in a herd of milch cows through their getting into an orchard and eating a large quantity of seedling apples and subsequently gaining admittance to a field of corn. When driven home and milked they showed no symptoms beyond a somewhat distended abdomen. After milking they were turned out to pasture for the night. The next morning seven or eight of them were found to be staggering in their walk, and the secretion of milk was completely arrested. The owner, upon the advice of a neighbor, administered a dose of pepper to each; in the afternoon of the same day seven of the eight affected were down, and the one still standing was very tympanitic; on attempting to raise the others they were found to be paralyzed and unable to regain the standing position. The above history was related to Mr. Moore on his arrival. On examining the cows he found that they were suffering from a profuse diarrhœa; the one suffering from tympanites was immediately relieved by the use of the trocar, and all received a dose of spirits of ammonia, turpentine, and linseed oil. On the following morning Mr. Moore saw them and found that the three that had regained their feet seemed much better, while four were still down. One old, emaciated cow died some two hours later. All then received a dose of soda, gentian, and nux vomica, and by four o'clock that afternoon the remaining three were on their feet and showed signs of returning appetite. The cows, not having passed any feces since the diarrhœa ceased, received a pint of linseed oil each, and subsequently a pound of magnesium sulphate, which, having the desired effect, all gradually regained their usual health. In the discussion which followed the suggestion was made that the cows might possibly have become intoxicated by fermentation of the apples in the stomach, and thus become paralyzed.

Mr. Killam then read a carefully prepared paper on "Breathlessness," in which he minutely described its various physiological aspects, also pointing out the effects of various altitudes on respiration. In high altitudes the number of respirations would of necessity be increased, owing to the partial pressure of the atmospheric oxygen diminishing as one ascended to such an extent that at an altitude above 8000 feet dyspnœa would be produced, and at one of 17,000 feet asphyxia might ensue, owing to this partial pressure of this atmospheric oxygen being less than in the pulmonary capillaries. After some discussion by the members Dr. Charles McEachran and Dr. Martin made some interesting remarks, and the meeting then adjourned.

B. A. SUGDEN,
Secretary.

